# Extracting Quality-of-Life Information of Patients Diagnosed With Breast Cancer From Health Care Online Forum Posts Using Open-Source Large Language Models: Algorithm Development and Evaluation Study

**DOI:** 10.2196/92716

**Published:** 2026-10-05

**Authors:** Karolina Hanna Czok, David Maria Schmidt, Brian Po-Han Chen, Deborah Kuk, Josh Feldman, Philipp Cimiano

**Affiliations:** 1 Center for Cognitive Interaction Technology, Faculty of Technology, Bielefeld University, Inspiration 1, Bielefeld, 33619, Germany, 49 521106 ext 2951; 2 Inspire, Arlington, VA, United States; 3 Teva Branded Pharmaceutical Products R&D LLC, Parsippany, NJ, United States; 4 Datavant, Phoenix, AZ, United States; 5 Semalytix GmbH, Bielefeld, Germany

**Keywords:** quality of life, patient-reported outcomes, breast cancer, online health care forums, social media analysis, large language models, AI

## Abstract

**Background:**

Quality-of-life (QoL) questionnaires are an established instrument designed to assess overall well-being and QoL of patients. They are important in predicting the outcome of the disease and understanding the needs of individual patients. However, their repeated collection imposes a substantial burden on both patients and clinical professionals. Many patients seek emotional support and mutual exchange in online communities for peer support, where they frequently share detailed descriptions of symptoms and treatment experiences, addressing topics covered in QoL questionnaires. The emergence of large language models (LLMs) uncovers potential for automatic extraction of relevant QoL information from patient-generated text.

**Objective:**

The aim of this study is to evaluate and compare various open-source LLMs and optimization approaches for automated extraction of QoL information from forum posts.

**Methods:**

The dataset consisted of 840 English-language posts from patients with breast cancer recruited on Inspire online communities, manually annotated with sentence-level text spans indicating whether and where posts contained information relevant to 53 QoL questions from standardized questionnaires. Eleven open-source LLMs were evaluated in a zero-shot setup under 2 input conditions: post-only and post with additional context. For the GPT-OSS-20B model, additional experiments assessed the impact of chain-of-thought prompting, instruction optimization, few-shot prompting, simultaneous all-questions prompting, and parameter-efficient fine-tuning. For correctly classified yes and no instances, the overlap between model-generated evidence and human-annotated spans was evaluated.

**Results:**

Across 11 evaluated LLMs, Qwen3-14B achieved the highest macro *F*_1_-score (0.59) in the zero-shot post-only setting. Providing additional context consistently reduced the performance of all models. Model size did not correlate with *F*_1_-score, with several midsized models (14B-30B) outperforming 70B models. For GPT-OSS-20B, chain-of-thought prompting, instruction optimization, and simultaneous all-questions prompting decreased performance. Bootstrap few-shot prompting with random search achieved slightly better performance than the baseline. Parameter-efficient fine-tuning with low-rank adaptation (LoRA) achieved the highest overall performance (0.71). Across all experiments, a strong class imbalance was observed, with models performing substantially better on the majority class “not in the text” than on the minority classes “yes” and “no.” Most classification errors occurred in semantically broad or ambiguous terms and the fallback question. For correctly predicted yes and no answers, model-generated evidence matched or partially matched human-annotated spans in 89% (42/47) of cases.

**Conclusions:**

Automated extraction of QoL information from patient-generated text using open-source LLMs remains a challenging task. While prompt optimization techniques failed to improve baseline zero-shot performance, parameter-efficient fine-tuning with LoRA significantly increased accuracy. However, current models still struggle to reliably detect explicit symptom expressions in heavily imbalanced data, too often predicting the majority class “not in the text.” Before such tools can be successfully integrated into clinical practice, future research must prioritize strategies to capture these minority-class signals.

## Introduction

Quality of life (QoL), as defined by the World Health Organization, encompasses physical, mental, and social well-being beyond the mere absence of disease [1]. Embedding QoL assessments into clinical practice can improve patient satisfaction, increase treatment engagement, help clinicians anticipate risk, and intervene earlier to address key impairments that matter to patients and influence prognosis [2,3]. Traditionally, the gold standard to assess QoL is fixed questionnaires, such as the European Organisation for Research and Treatment of Cancer (EORTC) Quality of Life Questionnaire-Core (QLQ-C30) for patients with cancer [4]. Despite the widely recognized value of QoL assessments, their repeated collection imposes a substantial burden on both patients and health care providers [5-8] and therefore remains underused in routine clinical care [3].

Medical information is available on the internet in many different forms, with the internet becoming the second most important source of information after physician consultations [9]. Patients facing a chronic or particularly serious illness have an increased need for information and discussion to exchange experiences with other people affected by the same disease [10,11]. Many associations offer forums dedicated to specific types of illnesses, particularly breast cancer, as places for expression, support, and a source of information, allowing users to belong to a community facing the same difficulties [12]. These discussions can contain useful information about a patient’s well-being, such as QoL information, as shown by Schmidt et al [13].

Breast cancer is the most commonly diagnosed cancer in women and the second most diagnosed cancer overall, with over 2.3 million new cases in 2022 [14]. However, approximately 92% of women diagnosed with breast cancer survive at least 5 years postdiagnosis [15]. Given the large and growing population of breast cancer survivors, research aimed at understanding their long-term needs is highly relevant [16]. The integration of QoL assessments into clinical practice has been shown to provide meaningful benefits for patients with breast cancer, offering them insights into their own care [17]. For clinicians, such information can support more individualized treatment decisions by highlighting which interventions may be most beneficial for specific patients based on their circumstances [18]. Lim et al [19] showed that certain QoL measures, such as better physical functioning, lower pain levels, and reduced appetite loss, are associated with improved survival outcomes in patients with cancer, including those with breast cancer. These findings highlight the value of considering self-reported measures alongside traditional clinical factors in treatment decisions, particularly for patients with breast cancer. Furthermore, women living with and surviving breast cancer report increasing use of a variety of social media platforms as part of their daily routine to manage ongoing care and psychosocial needs [20]. Consequently, a substantial amount of potentially valuable patient-generated data exists; however, it remains largely underused in clinical and research settings.

Recent advancements in AI, particularly the emergence of large language models (LLMs), offer promising avenues to overcome the data collection bottleneck by extracting QoL information from free-text narratives and mapping it to standardized questionnaire items. LLMs have demonstrated promising performance across a range of clinical natural language processing (NLP) tasks involving unstructured text, such as information extraction [21-23] and summarization [24]. The use of locally hosted, open-source LLMs enables compliance with data privacy requirements that are critical for the deployment of health care applications.

Despite these advances, the application of LLMs to extract QoL information from patient-generated text remains underexplored and, to the authors’ best knowledge at the time of writing, no prior studies have directly investigated this problem.

The objective of this study is to address the following research questions (RQs):

- RQ1: Among open-source LLMs, which model performs best in a zero-shot setting for extracting QoL questionnaire answers from health forum posts?
- RQ2: How do different prompting and optimization strategies (few-shot prompting, instruction optimization, chain-of-thought (CoT) reasoning, simultaneous all-questions prompting, parameter-efficient fine-tuning with low-rank adaptation [LoRA]) influence performance?
- RQ3: To what extent can LLMs generate textual evidence that aligns with human-annotated spans supporting yes and no predictions?
- RQ4: Which QoL questionnaire items are most prone to misclassification and what types of errors occur most frequently?

By answering these questions, this study aims to evaluate the potential and limitations of using open-source LLMs on patient-generated forum data as a low-burden and low-cost complement to traditional QoL assessment.

## Methods

### Overview

The study evaluated the feasibility of extracting QoL information from forum posts using LLMs through four phases: (1) model comparison: comparing 11 open-source LLMs of varying sizes (8B to 70B) in post-only and post+context settings; (2) optimization evaluation: assessing CoT prompting, instruction optimization, few-shot prompting, simultaneous all-questions prompting, and parameter-efficient fine-tuning with LoRA for the chosen midsized model; (3) evidence generation: prompting the previously chosen model to provide textual evidence supporting every correct yes and no prediction and comparing this evidence with human-annotated spans; (4) error analysis: calculating error counts per question and per error type. The goal of this framework is to provide a comprehensive assessment of LLMs’ capabilities in extracting QoL information and to identify potential limitations.

### Dataset

#### Overview

This study uses the dataset introduced by Schmidt et al [13], which comprises three main parts: (1) posts from the Inspire online communities written by users who completed QoL questionnaires; (2) sentence-level annotations of the posts indicating where specific QoL questions are addressed; (3) gold-standard responses to QoL questionnaires, specifically the EORTC QLQ-C30 and 23-item European Organization for Research and Treatment of Cancer Quality of Life Questionnaire - Breast Cancer Module (EORTC QLQ-BR23), provided by the participating users. The EORTC QLQ-C30 is a 30-item questionnaire assessing general QoL in patients with cancer [4], while the QLQ-BR23 consists of 23 questions focused on breast cancer–specific aspects [25]. It is a supplementary module to be used in conjunction with the EORTC QLQ-C30. Inspire is a large online health community consisting of patients and caregivers across a wide range of medical conditions, including cancers. The platform is designed to facilitate open discussion of sensitive health-related topics and to support peer-to-peer interaction among individuals with similar health journeys. Posts are unstructured and vary from short replies to long-form narratives, typically describing symptoms, emotional states, treatment experiences, side effects, and everyday life challenges. Importantly, these posts are independently authored and not written in response to the QoL questionnaires. Instead, they reflect naturally occurring patient experiences.

The dataset contains 20,204 English-language forum posts and comments from 2006 to 2024, with 580 posts and comments originating within the 6 months preceding the end of the study period. In total, 2683 posts and comments were manually annotated at the sentence level. Among these, 613 contain at least 1 QoL-related annotation. During annotation, trained annotators identified sentences containing information relevant to individual QoL questionnaire items and linked them to the corresponding questions. Annotation was performed by 3 annotators for the most recent 6-month period and 2 annotators for the preceding 18-month period, following the annotation protocol defined in Schmidt et al [13].

The authors have demonstrated that these annotated posts contain substantial information relevant to QoL assessment, showing that user-level questionnaire responses could be predicted from the coded data with an *F*_1_-score of approximately 0.70. The 5 most frequently answered questions in the coded data were: “Did you feel ill or unwell?,” “Did you worry?,” “Have you had pain?,” “Did you feel tense?” and “Were you limited in doing either your work or other daily activities?” The question “Did you feel ill or unwell?” was used as a fallback label for symptoms that do not fit into any other question. In the original study, the relationship between forum content and questionnaire responses was analyzed by comparing annotations derived from posts within different temporal windows preceding questionnaire completion, including the most recent 6 months and the most recent 24 months. The authors found that QoL-relevant information contained in forum posts shows substantial agreement with questionnaire responses across both time windows, indicating that patient-generated content can provide meaningful information about QoL over extended periods [13].

In this paper, the EORTC QLQ-C30 and EORTC QLQ-BR23 questionnaires are used exclusively as a source of QoL questions, and the sentence-level annotations are treated as ground truth indicators of whether and where a given post contains information relevant to a particular question. The task is performed at the post level and does not involve predicting full questionnaire outcomes for users based on their complete posting history. Instead, it focuses on determining whether individual QoL questions can be answered based on a single post using LLMs. The task is framed as a 3-way post-level classification. For each of the 53 questionnaire items, a model must assign 1 of 3 distinct target labels: yes (the post confirms the presence of the symptom or experience), no (the post explicitly negates it), or not in the text (the item is completely unaddressed). This formulation enables fine-grained analysis of where specific QoL information is expressed and allows verification of whether the predicted information is correct, as well as characterization of different types of errors made by LLMs. In addition, it allows assessment of whether LLMs can reproduce human-annotated evidence spans for this task.

For model training and evaluation, the dataset was preprocessed by excluding posts used in a pilot annotation process, posts that described the conditions of individuals other than the posters themselves, such as relatives or friends, and posts specifically referring to distant past events. While posts describing experiences of a third person often contained health-related information, they were not directly relevant to the QoL of the person posting and were thus excluded from the final dataset. The remaining data were split using random sampling into 80% (672/840 posts) train, 10% (84/840 posts) validation, and 10% (84/840 posts) test sets, with the test set used for model evaluation and error analysis. Due to the multilabel nature of the dataset and the long-tail distribution of QoL annotations per post, exact stratification of all label combinations across train, validation, and test splits was not feasible. Many label combinations occur only a few times, which prevents reliable proportional allocation without overfitting the split design. To ensure comparability across splits, we instead verified that the marginal distributions of labels are consistent. In particular, the proportion of posts containing at least 1 QoL annotation is similar across train, validation, and test sets, and the overall label frequency distribution follows the same long-tailed pattern in all splits. Approximately half of the posts in each split contain no QoL annotations (train: 336/672, 50%; validation: 42/84, 50%; and test: 43/84, 51%).

#### Corpus Statistics and Class Imbalance

To provide a granular view of the dataset’s composition and highlight the inherent difficulty of the task, we analyze the global distribution of the target labels (“yes,” “no,” and “not in the text”) across the test set. As shown in Table 1, the resulting dataset exhibits an extreme class imbalance, characterized by massive sparsity of “yes” and “no” labels.

**Table 1.** Global class distribution across the test set.

Target label	Total instances and percentage of corpus, n (%)
Yes	59 (1.33)
No	9 (0.20)
Not in the text	4384 (98.47)

This highly skewed distribution reflects a real-world challenge in clinical text mining from patient-generated health data: while a patient’s overall posting history across months may contain rich QoL insights, any individual forum post or comment typically focuses only on a narrow subset of immediate concerns or symptoms. Consequently, the overwhelming majority of standardized questionnaire items remain unaddressed (not in the text) within a single post. Additionally, patients are naturally more inclined to report the presence of a symptom or experience rather than explicitly state its absence, particularly when responding to a main thread, which accounts for the higher frequency of “yes” labels relative to “no” labels.

#### Dataset Schema

The dataset follows a hierarchical structure linking posts, sentence-level evidence spans, and QoL questionnaire items. Each data instance consists of a forum post (or comment) paired with zero or more annotations that map text spans to specific QoL questions.

At the lowest level, each record contains:

- main_post: the main post or comment that the user is replying to (may be null).
- content: the original forum post text written by a user textual content associated with the same thread or reply context.
- labels: a list of annotated QoL mappings for the post.

Each element in labels links a span of text to a specific questionnaire item and contains:

- question: the QoL questionnaire item (from EORTC QLQ-C30 or EORTC QLQ-BR23).
- text: the exact extracted span from the post that provides evidence for the question.
- begin, end: character offsets of the annotated span within the post.
- exactmatch: whether the span exactly answers the question.
- negative: whether the span indicates a negative statement.

These schema attributes map directly to the target classification labels. If a question contains no associated annotation span for a post, its target label is “not in the text.” If an annotation span exists and the negative attribute is false, the target label is “yes.” If an annotation span exists and the negative attribute is true, the target label is “no.” A single post may contain multiple labels if it includes evidence for multiple QoL aspects or no labels if no QoL question is answered by the post.

#### Participants

The focus of the dataset is patients with breast cancer who have been recruited from Inspire, specifically from the “Breast Cancer” and “Advanced Breast Cancer” communities. The inclusion criteria were (1) females with a breast cancer diagnosis, (2) age 18 years or older, (3) residency in the United States, and (4) at least 1 post or comment in the respective Inspire communities.

In total, 134 participants participated in the study, of which 11 did not have at least 1 post, resulting in 123 participants meeting the inclusion criteria. The average age of the participants was 68.2 (SD 11.5) years, with an average age of 55.7 (SD 12.6) years at the time of the diagnosis. Moreover, at the time of the survey, the average number of years that passed since the diagnosis of the patients was 13.1 (SD 9.4).

### Selection of LLMs and Experimental Environment

One of the foremost ethical concerns in AI health care is the security and confidentiality of sensitive health data [26]. While commercial LLMs such as GPT (OpenAI) or Gemini (Google) offer powerful linguistic capabilities to support various medical tasks, their cloud-based nature raises significant privacy challenges [27,28]. Using locally hosted, open-source models can provide a privacy-preserving alternative, adhering to health care regulations and ensuring that sensitive medical information remains within the local environment [29]. In the first part of the study, 11 open-source base (ie, noninstruct) LLMs representing diverse model families were selected: GPT-OSS (20B) [30], Qwen3 (14B, 30B) [31], Gemma3 (12B, 27B) [32], DeepSeek-R1 (14B, 32B, 70B) [33], Llama 3.1 (8B, 70B) [34], and Phi-4 (14B) [35]. The model sizes ranged from 8B to 70B parameters. Models were run locally via the Ollama [36] framework, avoiding transmission of data to cloud-based APIs. Experimental pipelines were implemented using the Declarative Self-improving Python (DSPy) [37] declarative framework, ensuring consistent input and output behavior (Textbox 1).

Textbox 1. **Expected input or output behavior specified as a Declarative Self-improving Python signature.**

**Input**

- “Content”: post or comment of a health care forum user to be investigated.
- “Main post”: context of the health care forum post, for example, the main post in a thread.

“Question” : question from the quality-of-life (QoL) questionnaire.

**Output**

- “Answer”: answer to the question, either:

- “not in the text”: the information relevant to the question is not mentioned.
- “no”: the post explicitly negates the content of the question.
- “yes”: the post explicitly confirms the content of the question.

### Zero-Shot Comparison of Models and Input Conditions

#### Overview

The first experiment examined baseline model performance in a zero-shot setting with and without adding context. Adding context means that, in each prompt, the investigated post is accompanied by the main post it refers to within a given thread, that is, the post to which the user is responding. For example, in the post+context setting, the prompt could consist of the reply “Yes, I experience it too” together with the main post “Does any of you have trouble with skin peeling after radiation?” In the postonly setting, only the reply “Yes, I experience it too” would be included in the prompt.

Each model was evaluated on the test set consisting of 84 posts, paired with the same set of 53 QoL questions from EORTC QLQ-C30 and EORTC QLQ-BR23 questionnaires, generating 4452 post-question predictions per model. For each post, the model was required to predict whether it contains information that would answer the question positively, negatively, or whether the information is not mentioned. An example of the prediction task is shown in Figure 1.

**Figure 1.**
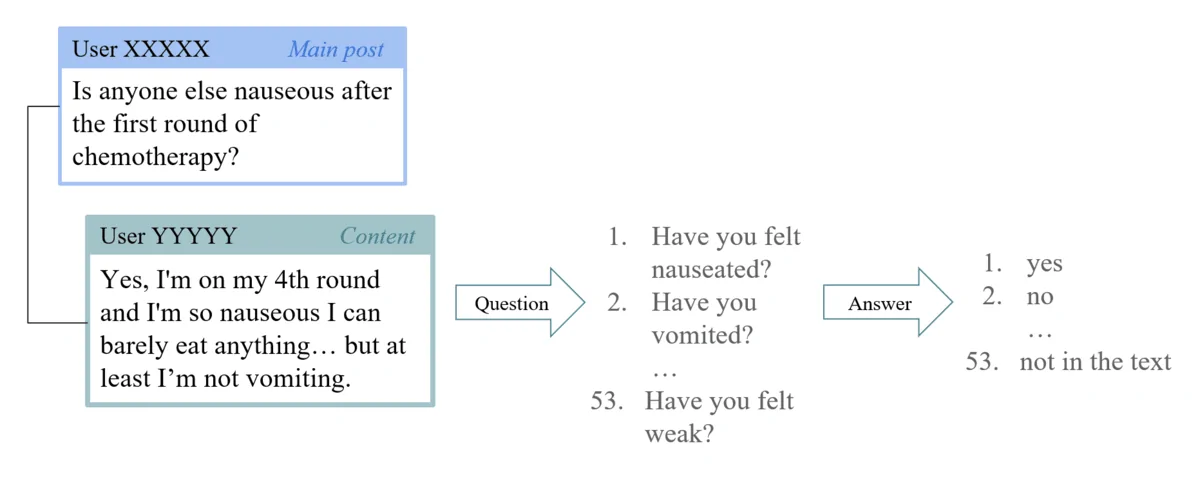
Example of patient-generated “content” with additional context “main post” evaluated on 53 “questions” from European Organisation for Research and Treatment of Cancer (EORTC) Quality of Life Questionnaire-Core 30 (QLQ-C30) and EORTC Quality of Life Questionnaire-Breast Cancer 23 (QLQ-BR23) questionnaires answered with “yes, no, not in the text.”

To check whether adding the main post from the corresponding discussion thread improves or degrades performance, each model was evaluated twice: once with and once without inclusion of “Main post” as additional context.

#### Evaluation

For every prediction, the model-generated label was compared against the human-annotated ground truth. However, the extreme class imbalance in this dataset creates a significant evaluation challenge. A naïve baseline model that always predicts “not in the text” would automatically achieve an accuracy of 98.47%. Evaluating models through aggregate metrics like weighted *F*_1_-score would provide a distorted view of their real performance. To ensure a transparent assessment, we evaluate all models using an unweighted macro *F*_1_-score (calculated as the arithmetic mean of the *F*_1_-scores for the 3 individual classes) alongside per-class breakdowns.

For each class (“yes,” “no,” “not in the text”), the *F*_1_-score is computed as the harmonic mean of precision and recall [38]:


$$F_{1}\text{=}\left(2\;\times \;Precision\;\times \;Recall\right)\text{/}\left(Precision\;+\;Recall\right)$$

The macro *F*_1_-score is then calculated as the arithmetic mean of the 3 classes:


$$Macro\;F_{1}=\left(F_{1,\;yes}+F_{1,\;no}+F_{1,\;not\_in\_the\_text}\right)/3$$

This ensured that each class contributed equally to the final score, preventing the most frequent “not in the text” class from dominating the performance assessment and masking minority class failures.

### Prompt Optimization and Fine-Tuning Experiments

#### Experiments Overview

Following the zero-shot evaluation, one of the midsized models was selected for all subsequent optimization experiments in this section. As this was a feasibility study, restricting experiments to a single model enabled controlled comparisons between optimization methods while minimizing confounding effects related to architectural differences and computational cost.

A series of optimization experiments were then conducted to assess whether different prompting strategies and lightweight fine-tuning approaches could improve performance in extracting QoL information. All experiments were run under the same task definition and input-output specification as the zero-shot baseline, without providing additional context (Main post). The only variations between experiments were the prompting strategies or additional training applied, ensuring that differences in performance reflected the effect of the optimization technique itself rather than changes in input structure or task formulation. For methodological consistency, all optimization methods were evaluated on the same test split of the dataset using the same macro *F*_1_-score as in the baseline experiment.

#### CoT Prompting

CoT is a prompting strategy that mimics the step-by-step thinking ability of humans. It is widely used to decompose multistep problems into intermediate steps, achieving improvement on many reasoning benchmarks by significantly improving the ability of LLMs to perform complex reasoning [39]. The experiment consisted of running the DSPy pipeline with the ChainOfThought predictor module, which instructed the model to generate a series of reasoning steps before producing the final label. The reasoning content was not evaluated and only the final classification contributed to the score.

#### Instruction Optimization With Multiprompt Instruction PRoposal Optimizer Version 2

Multiprompt Instruction PRoposal Optimizer Version 2 (MIPROv2) [40] is a prompt optimizer capable of optimizing both instructions and few-shot examples jointly in 3 steps:

- Bootstrapping few-shot examples, by randomly sampling from the training set and running them through the program. If the output is correct, the example is kept as a candidate. Otherwise, the optimizer tries another example until the specified number of few-shot example candidates is curated.
- Generating instruction candidates using a composite prompt that included (1) a summary of the training dataset properties, (2) a summary of the program code and target predictor, (3) previously bootstrapped few-shot examples, and (4) a randomly sampled tip for generation (eg, “be creative” or “be concise.”).
- Finding the best combination of few-shot examples and instructions using Bayesian optimization. Sets of prompts are evaluated over a validation set for a specified number of trials, with the best set being returned at the end.

The experiment was performed with 2 variants:

- Zero-shot MIPROv2, which optimizes the instruction program without providing any demonstrations.
- Few-shot MIPROv2, which optimizes the instruction by also including bootstrapped examples.

Both variants were configured using the medium optimization mode provided by DSPy’s MIPROv2 framework. This setting was selected to evaluate optimization behavior under a standard configuration, rather than to exhaustively search the optimization space.

#### Few-Shot Prompting

To evaluate whether a more advanced bootstrapping method would improve the effect of adding few-shot examples to the unoptimized instruction, the next experiment applied DSPy’s bootstrap few-shot prompting with random search. This optimization method generates a set of candidate few-shot prompts by combining demonstrations directly from the training set with additional bootstrapped examples produced by a teacher model. It then performs a randomized search over multiple candidate prompt configurations and selects the best-performing program [41].

The configuration was set to include up to 5 labeled and 5 bootstrapped demonstrations per prompt and to evaluate 3 candidate programs. This setting was chosen to limit computational cost while allowing the optimizer to explore multiple prompt configurations within the scope of this feasibility study.

#### Fine-Tuning With LoRA

Fine-tuning is a process of adapting a pretrained model for specialized tasks or domains, by training it on a custom dataset [42]. In the context of the medical domain, it enables the model to access specialized clinical terminology, often absent in general language used for pretraining [43,44].

LoRA presents a parameter-efficient approach to fine-tuning LLMs, which enables targeted training without the need to modify the entire model [45]. While most of the parameters are fixed, only a small number of parameters are trained to adapt to a new task. This approach requires significantly fewer computational resources than full fine-tuning and avoids altering the base model, thus preserving the pretrained knowledge [46].

Training data consisted of all labeled training examples from the QoL classification dataset. To reduce class imbalance, the minority classes (“yes” and “no”) were balanced through random oversampling to match the number of “not in the text” examples in the training set. During fine-tuning, the model was trained to predict only the target answer, while the prompt tokens were masked from the loss computation. Parameter-efficient fine-tuning was performed for 3 epochs using LoRA with a learning rate of 2×10^−5^, an effective batch size of 8, and brain floating point with 16 bits (bfloat16) precision. Only the LoRA adapter parameters were updated, while all base model weights remained fixed. After training, the adapter was saved and used to evaluate the model on the test set under the same conditions as all other experiments.

#### Simultaneous All-Questions Prompting

To investigate the impact of query structure on model performance, simultaneous all-questions prompting was evaluated. In the standard setup, models were queried sequentially, evaluating a single post against a single question at a time. In this alternative configuration, all 53 QoL questions were presented simultaneously within a single prompt. This experiment allows us to determine whether concurrent information extraction increases the models’ performance compared to single-question prompting.

### Generation of Textual Evidence and Comparison With Human Annotations

Due to the complexity of medical data, producing a comprehensive and accurate set of text spans corresponding to specific medical entities often requires involvement of multiple experts with specialized knowledge. The process is therefore costly and resource-intensive, which raises the question of whether LLMs could be used to streamline the process of annotating medical data [47,48].

The following experiment assessed whether LLMs are capable of generating textual evidence supporting their predictions, a task that could be later extended to producing annotations for the QoL information extraction dataset. For all correctly classified “yes” and “no” predictions in the best optimization setting, the chosen model was prompted to provide the exact sentence from the post that justified the answer. The evidence was compared against human-annotated spans contained in the original dataset and categorized into:

1. Exact matches - the model-generated evidence span is identical to the corresponding human-annotated text span.
2. Partial matches - the model-generated evidence was fully contained within the human-annotated text span, or vice versa, and the token overlap was at least 50%.
3. No matches - the model-generated evidence did not meet the partial match criterion, either because the token overlap was below 50% or the evidence and annotation were not contained within the same sentence.

Table 2 illustrates the three match categories using examples of model-generated evidence and the corresponding human-annotated spans.

**Table 2.** Example of generated evidence for correct “yes” predictions compared to human-annotated spans.

Question	True answer	Predicted answer	Human annotation	LLM^a^ evidence	Type of match
Have you felt nauseated?	yes	yes	The nausea is much worse during my second chemo cycle.	The nausea is much worse during my second chemo cycle.	Exact match
Were you tired?	yes	yes	Lately I’ve been feeling exhausted most days	Lately I’ve been feeling exhausted most days, especially in the afternoons	Partial match
Did you feel ill or unwell?	yes	yes	I stopped taking tamoxifen because of the side effects	Some days I just feel unwell and overwhelmed by everything	No match

a LLM: large language model.

Token overlap was computed as the proportion of tokens shared between the model-generated evidence and the human-annotated span relative to the longer of the 2 spans.

The proportion of exact and partial evidence matches, relative to the total number of evaluated evidence, was calculated to assess how closely model-generated evidence corresponded to human-identified spans of relevant information.

### Error Analysis and Question-Level Mispredictions

The final experiment examined error distribution across QoL questions and the types of errors occurring:

- False positives: predicting yes or no when no relevant information was present.
- False negatives: predicting not-in-text when relevant information is present.
- Label confusion: inverting yes ↔ no.

The predictions for QoL questions with the highest misclassification rates were manually analyzed to identify systematic weaknesses, such as ambiguity or semantic overlap (eg, fatigue vs need to rest).

### Ethical Considerations

The study has been approved by the Ethics Committee of Bielefeld University under application number 2023‐216-W1. Informed consent was obtained from all participants to analyze their posts and comments for this study. The first 100 respondents received a US $20 gift card. The collected data is stored in encrypted form and available only to the authors of the study. All models used in the study are open-source and run in a local environment; therefore, no data have been transferred to the cloud servers.

## Results

### Baseline Model Comparison in the Zero-Shot Setting

Table 3 summarizes the macro *F*_1_-scores across all 11 evaluated models. Qwen3-14B achieved the highest overall performance in both experimental configurations, reaching a macro *F*_1_-score of 0.59 in the post-only setting and 0.56 when context was included. Removing context consistently improved performance of nearly all models, with Δ*F*_1_ (denoting the increase in macro *F*_1_-score after removing context) varying between 0.03 and 0.11. The results do not point to a single model family as universally superior for this task; however, Qwen3 and GPT-OSS families performed slightly better than the alternatives.

**Table 3.** Comparison of macro *F*_1_-scores with 95% CIs across 11 open-source LLMs^a^ for zero-shot QoL^b^ information extraction with and without post context with per-class breakdown in the brackets.

Model	Params	Post only				Postcontext				ΔF_1_^c^
		Macro F_1_-score (95% CI)	Class breakdown			Macro F_1_-score (95% CI)	Class breakdown			
			Yes	No	Not in the text		Yes	No	Not in the text	
deepseek-r1	70B	0.51 (0.46‐0.55)	0.42	0.07	0.98	0.46 (0.32‐0.50)	0.34	0.08	0.97	0.05
llama3.1	70B	0.48 (0.44‐0.53)	0.35	0.13	0.98	0.43 (0.40‐0.45)	0.25	0.06	0.96	0.05
deepseek-r1	32B	0.51 (0.47‐0.54)	0.47	0.07	0.97	0.43 (0.40‐0.45)	0.32	0.03	0.93	0.08
qwen3	30B	0.52 (0.48‐0.55)	0.48	0.09	0.98	0.52 (0.48‐0.57)	0.48	0.11	0.98	0
gemma3	27B	0.51 (0.44‐0.58)	0.34	0.22	0.98	0.47 (0.42‐0.52)	0.27	0.18	0.97	0.04
gpt-oss	20B	0.58 (0.48‐0.66)	0.42	0.33	0.99	0.54 (0.47‐0.60)	0.35	0.28	0.98	0.04
qwen3	14B	0.59 (0.51‐0.66)	0.5	0.29	0.99	0.56 (0.49‐0.62)	0.46	0.22	0.99	0.03
phi4	14B	0.52 (0.45‐0.59)	0.42	0.16	0.99	0.41 (0.39‐0.44)	0.26	0	0.98	0.11
deepseek-r1	14B	0.49 (0.45‐0.52)	0.42	0.07	0.98	0.45 (0.42‐0.49)	0.31	0.08	0.97	0.04
gemma3	12B	0.45 (0.42‐0.49)	0.34	0.05	0.98	0.41 (0.39‐0.44)	0.24	0.03	0.96	0.04
llama3.1	8B	0.36 (0.34‐0.38)	0.21	0.02	0.85	0.32 (0.31‐0.33)	0.11	0.01	0.84	0.04

a LLM: large language model.

b QoL: quality-of-life.

c Δ*F*_1_ represents the incremental change in macro *F*_1_-score when context is removed, calculated as:$\Delta F_{1}=Macro\;F_{1,post\_only}-Macro\;F_{1,post\_with\_context}$.

A granular analysis of the per-class metrics reveals the underlying difficulty of the task and the reason for the overall poor performance. While all models excelled at predicting the majority “not in the text” label with nearly all scores exceeding 0.96, performance collapsed on the minority classes. The highest recorded *F*_1_-score for the “yes” class was only 0.50, while performance on the “no” class was even lower, at just 0.33.

Figure 2 illustrates the relationship between model size and the macro *F*_1_-score for 2 different input configurations: post+context (square) and post-only (circle). There is not a simple linear relationship between model size and performance, with several midsized models (14B-30B) outperforming 70B models. Performance generally improves from smaller to midsized models and drops again for larger models.

**Figure 2.**
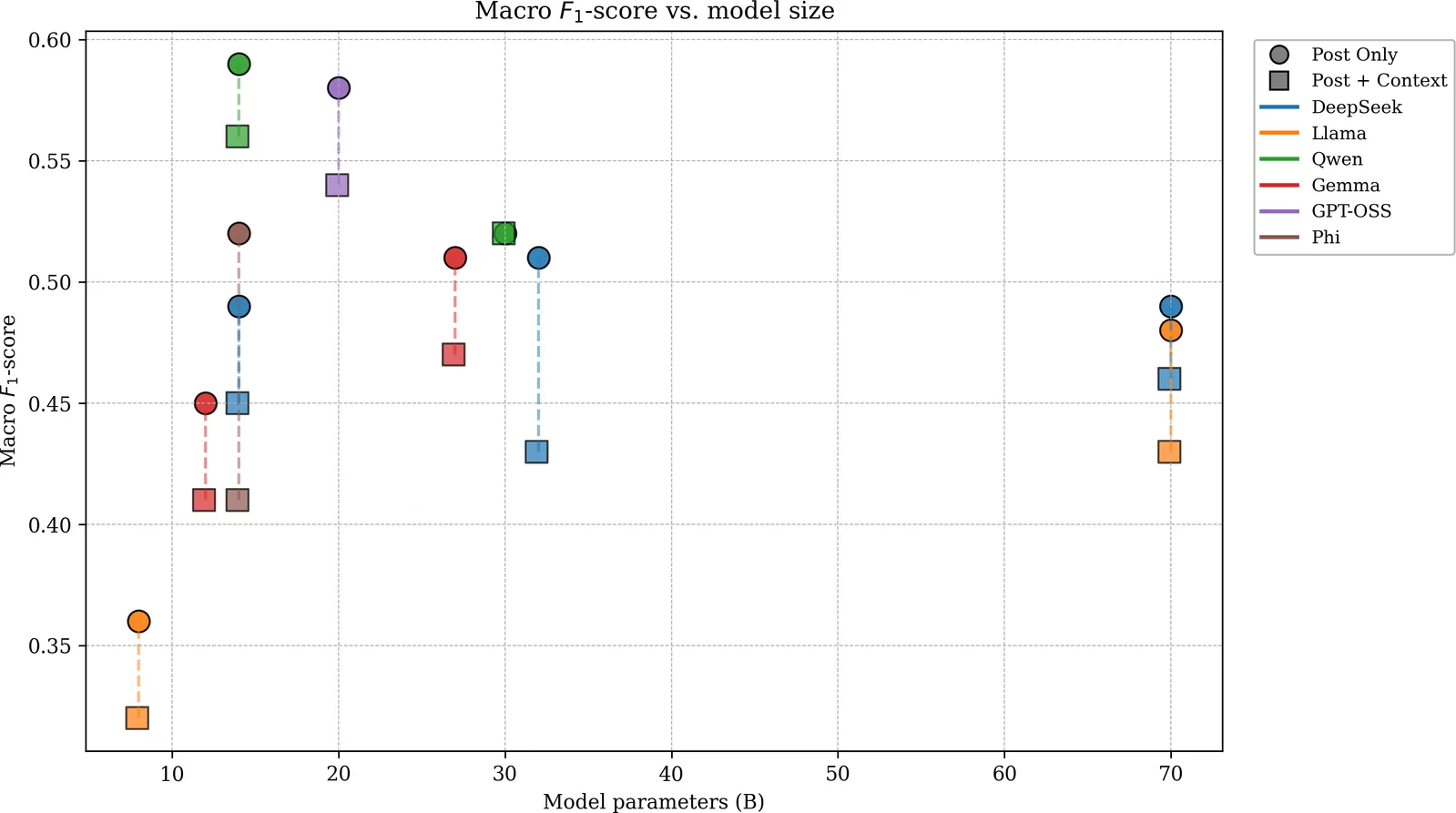
Macro *F*_1_-score as a function of model size by family for 2 input settings. Every model family is represented by a different color. Vertical dashed lines represent the “performance penalty” from adding context. B: billion.

For all subsequent optimization and fine-tuning experiments, GPT-OSS-20B in the post-only input setting was selected as a representative midsized architecture. It demonstrated one of the strongest zero-shot baseline performances among all evaluated models, serving as the baseline from which we would measure the impact of applying optimization strategies.

### Comparison of Prompt Optimization and Fine-Tuning Strategies

Table 4 summarizes the performance of GPT-OSS-20B across the different optimization strategies. Contrary to expectations, CoT prompting resulted in lower performance than the plain prediction baseline, decreasing the macro *F*_1_-score from 0.58 to 0.52. Similarly, both MIPROv2 optimization strategies failed to outperform the baseline, achieving macro *F*_1_-scores of 0.56 in the zero-shot setting and 0.54 in the few-shot setting. Bootstrap few-shot optimization with random search demonstrated the strongest performance among the prompting-based approaches, reaching a macro *F*_1_-score of 0.60, representing only a small improvement over the baseline. Prompting all 53 QoL questions simultaneously resulted in the poorest performance (macro *F*_1_-score=0.46), indicating that increasing the number of questions within a single prompt negatively affected extraction quality. In contrast, parameter-efficient fine-tuning with LoRA substantially improved predictive performance, achieving the highest overall macro *F*_1_-score of 0.71.

**Table 4.** Comparison of GPT-OSS-20B performance with 95% CIs under different optimization strategies.

Optimization strategy	Macro *F*_1_-score (95% CI)	Class breakdown		
		Yes	No	Not in the text
None	0.58 (0.48‐0.66)	0.42	0.33	0.99
Chain-of-thought prompting	0.52 (0.47‐0.58)	0.46	0.13	0.99
MIPROv2^a^ Zero-shot	0.56 (0.49‐0.62)	0.42	0.27	0.99
MIPROv2 Few-shot	0.54 (0.47‐0.62)	0.46	0.21	0.99
Bootstrap few-shot with random search	0.60 (0.50‐0.69)	0.49	0.32	0.99
All-questions prompting	0.46 (0.42‐0.50)	0.34	0.05	0.98
Fine-tuning with LoRA^b^	0.71 (0.58‐0.80)	0.68	0.47	0.99

a MIPROv2: Multiprompt Instruction Proposal Optimizer Version 2.

b LoRA: low-rank adaptation.

### Textual Evidence Alignment With Human Annotations

Table 5 summarizes the alignment of model-generated textual evidence with human annotations. Out of all 4452 predictions, 47 were correctly classified with a “yes” or “no” label. In 42 of 47 (89%) predictions, the model-generated evidence either exactly matched or partially matched the human-annotated span. Exact matches were observed in 20 cases (n=47, 42%) and partial matches in 22 cases (n=47, 47%). Only 5 cases (n=47, 11%) were classified as no match for 4 different questions: “Did you feel ill or unwell?” (2 cases) and “Have you had pain?,” “Have you had any pain in the area of your affected breast?,” and “Has your physical condition or medical treatment interfered with your social activities?” (1 case each).

**Table 5.** Alignment between model-generated and human-annotated evidence spans for correctly classified yes or no predictions.

Match type	Values, n (%)
Exact match	20 (42)
Partial match	22 (47)
No match	5 (11)

### Misclassification Patterns Across QoL Questions

Analysis of model errors in the baseline setting revealed that a primary difficulty occurred when deciding whether relevant information was present in the text. Approximately 76% (85/112) of misclassifications constituted the model predicting a positive or negative answer for questions with the correct label “not in the text.” The remaining 24% (27/112) of errors occurred when the model predicted “not in the text” despite the presence of information supporting a “yes” or “no” answer. No cases were observed in which the model generated a reversed answer (ie, predicting “yes” instead of “no” or vice versa).

A less prominent pattern was observed for the LoRA fine-tuned model. 40% (20/50) of LoRA errors corresponded to predicting “not in the text” despite evidence supporting a “yes” or “no” answer, while 58% (29/50) corresponded to predicting a positive or negative answer when the correct label was “not in the text.” Only 2% (1/50) of errors involved inversion of “yes” and “no” labels.

Errors were unevenly distributed across QoL questions, as shown in Table 6. The fallback question “Did you feel ill or unwell?” was the most error-prone question in the dataset, with 22 errors (26% error rate). The second and third most misclassified questions were “Did you worry?” with 10 errors (12% error rate) and “Have you had pain?” with 9 errors (11% error rate).

**Table 6.** Top 10 QoL questionnaire items with the highest number of incorrect predictions.

Question	Error count
Did you feel ill or unwell?	22
Did you worry?	10
Have you had pain?	9
Were you limited in doing either your work or other daily activities?	6
Did you need to rest?	6
Were you worried about your health in the future?	5
Were you tired?	5
Did pain interfere with your daily activities?	4
Have you felt weak?	4
Did you feel depressed?	3

## Discussion

### Principal Results

This study investigated whether open-source LLMs can be used as a supplementary tool for extracting QoL information from online forum posts, using a breast cancer forum as an example, in a way that aligns with standardized questionnaire responses. It further explored which model and optimization technique may be best-suited for this task, assessed whether LLMs can potentially reproduce human annotations, and investigated possible challenges based on the types of errors occurring for specific QoL items.

Open-source LLMs demonstrated poor ability to identify QoL-relevant information from patient-generated text, achieving macro-averaged *F*_1_-scores between 0.32‐0.56 in a zero-shot setting with added context and 0.36‐0.59 without added context. This suggests that QoL information extraction remains a challenging task for current open-source LLMs without task-specific training. Overall performance depends on the model family and size; careful choice of model may therefore be a crucial starting point in the methodology design. Models from the GPT-OSS and Qwen families demonstrated higher potential for successfully extracting QoL information than other models. Notably, increasing the model size did not result in the highest scores, suggesting that increased parameter count alone does not guarantee better performance for this task. From a practical perspective, the stronger performance of midsized models compared to larger 70B models may be especially advantageous in clinical practice, as these models typically require fewer computational resources and less specialized hardware for deployment. However, inference time and resource consumption were not evaluated in this study and remain important directions for future work.

The analysis of per-class *F*_1_-scores further reveals a strong imbalance in performance across classes, with models achieving substantially higher performance on the majority class “not in the text” compared to the minority classes “yes” and “no,” which highlights a systematic weakness when using zero-shot LLMs for QoL information extraction from health care forum posts. From a clinical safety perspective, the models’ strong performance on the “not in the text” label is beneficial. In health care environments, minimizing false positives prevents the system from hallucinating nonexistent symptoms, which could lead to inappropriate clinical conclusions. However, the primary objective of deploying LLMs in this context is the successful extraction of explicit “yes” and “no” signals. In a real-world clinical monitoring workflow, failing to detect instances where a patient reports severe treatment side effects or acute emotional distress introduces critical false negatives. Missing these signals directly compromises the ability to track a patient’s QoL, making a zero-shot extraction framework insufficient for practical clinical deployment.

Providing additional context (the main post to which the investigated post refers) consistently led to decreased performance, suggesting that additional conversational context may introduce noise rather than clarifying information for QoL information extraction. Confusion matrices for the post-only and post+context settings (Figure 3) show that including context increased the number of misclassifications, particularly by incorrectly assigning “yes” or “no” labels to posts containing no information about the symptoms. This observation was supported by a manual analysis of prediction errors, which showed that approximately 37% of errors in the post+context setting resulted from confusion between the target post and the main post. For example, in 1 instance the main post contained the statement “I've been on CO2 for 2 years & never felt depressed. Suddenly I'm having terrible anxiety attacks. I'm barely eating or sleeping...,” while the corresponding target post consisted of another user’s recommendation to seek therapy for anxiety. For the question “Have you had trouble sleeping?,” the correct label was “not in the text,” as the question referred to the author of the target post rather than the person speaking in the main post. However, when conversational context was provided, the model predicted “yes,” suggesting that it relied on information from the context instead. Adding context should therefore be avoided for this task or the methodology should be carefully designed to prevent confusion, particularly if future studies suggest that removing context results in loss of relevant information.

**Figure 3.**
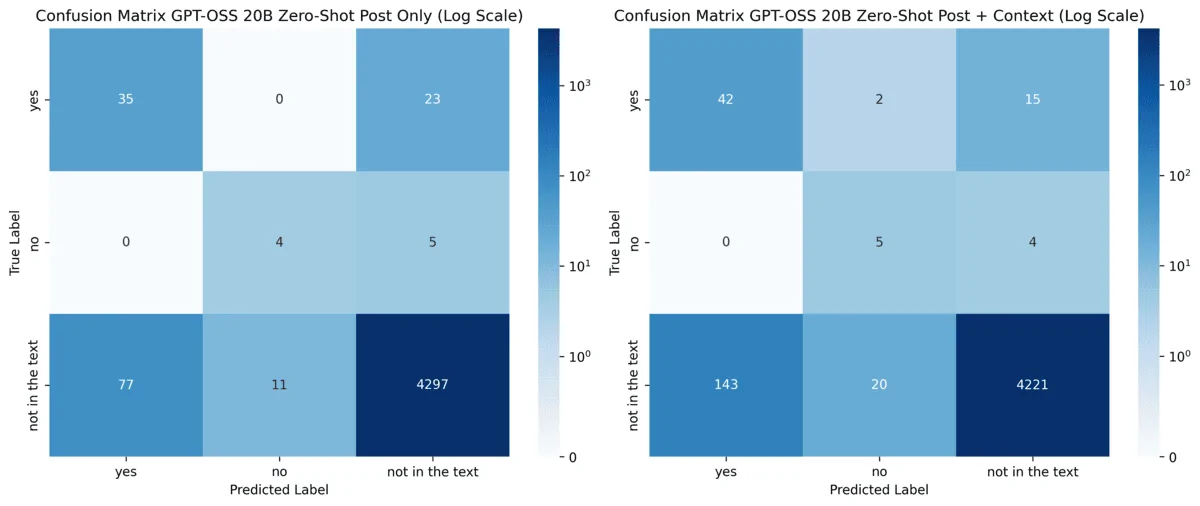
Confusion matrices for zero-shot quality of life (QoL) classification in post-only and post+context settings.

The evaluation of prompting-based optimization strategies applied to GPT-OSS-20B showed that prompt engineering methods provide only limited improvements over the baseline. CoT prompting resulted in a performance decrease compared to the plain zero-shot baseline, which aligns with findings by Liu et al [49] that CoT can reduce performance on tasks where thinking makes humans worse-here reflected as “overthinking” and assigning QoL questions to indirect symptoms. The qualitative examples in (Table 7) provide evidence for this effect, showing cases where the model shifts from direct symptom interpretation to more speculative reasoning paths, which leads to incorrect final predictions. Instruction optimization using MIPROv2 resulted in a slight performance decrease compared to the baseline in both zero-shot and few-shot settings. Bootstrap few-shot prompting with random search achieved the best results among prompt-based methods; however, the improvement over the baseline remained marginal. A possible explanation for the limited effectiveness of prompt-based optimization techniques is that the task requires fine-grained matching between patient-generated texts and specific QoL questions, while prompt optimization methods mainly improve general instruction-following or reasoning behavior. In particular, methods such as MIPROv2 optimize a single global prompt over the entire dataset, which may not be well suited for this setting due to the high variability across the 53 QoL questions, which differ in the symptoms they target and in how explicitly they are expressed in patient language. As a result, a single optimized prompt may not improve performance consistently across all questions and can even introduce more general or abstract reasoning patterns that are not helpful for identifying symptom mentions. While carefully selected few-shot demonstrations can provide some task-specific guidance, they are still not sufficient to cover the diversity of both patient-generated posts and QoL question formulations.

**Table 7.** Examples of chain-of-tCoT reasoning errors and resulting misclassifications.

Post	Question	True answer	Predicted answer	Reasoning
Everyone’s descriptions above of side effects mimic my experiences with fatigue and esp the metallic taste. Nausea meds were pretty ineffective compared to now. That being said, my body reacted about the same to each treatment and I had 9 mos of treatment. I knew that by day 4 after the treatment I would be feeling better and could eat more. Knowing how my body would react helped me handle the effects. Obviously, the drugs worked for me and the cancer had a very low 5-year survival rate, so I have been blessed.	Have you felt weak?	not in the text	yes	The user explicitly mentions experiencing fatigue as a side effect of the chemotherapy. Fatigue is commonly associated with feeling weak. Therefore, the post indicates that the user has felt weak.
I had 5 weeks of radiation that covered the entire breast and under the arm where the sentinel node biopsy was done. I went Monday through Friday, and other than the fatigue and, of course, the terrible redness and discomfort from the treatment, it was actually okay.	Do you need to stay in bed or a chair during the day?	not in the text	no	The post states that the user underwent 5 weeks of radiation, attending sessions Monday through Friday. The user mentions fatigue and discomfort but indicates that overall it was “actually okay.” This implies that the user was not confined to bed or a chair during the day; they were able to attend treatment sessions regularly. Therefore, the post answers the question by indicating that staying in bed or a chair during the day is not necessary.
Hello all, Does anyone have any ideas regarding low calcium levels in 24-hour urine testing? My Calcium Oxalate Saturation was well below normal, and my Calcium Phosphate and Calcium/Creatinine Ratio were also low. Chloride was lower than normal and PH was higher than normal. I, of course, will ask my endo doc when I see her on October 1st but I have tried to research reasons for these low levels and can not find any answers. I have been diagnosed with osteopenia. Does anyone have any ideas regarding these results?	Were you worried about your health in the future?	not in the text	yes	The user is actively seeking explanations for abnormal lab results and expresses concern about their health by asking for ideas and noting they will consult a doctor. This indicates worry about their future health.

In contrast, parameter-efficient fine-tuning with LoRA resulted in a substantial improvement in performance compared to all prompt-based methods. This suggests that adapting model parameters is more effective for this task than modifying prompts, likely because the model is able to learn task-specific patterns directly from the training data instead of relying on general instruction templates. In particular, fine-tuning allows the model to better capture subtle differences between similar QoL questions and to adjust to the specific language used in patient-generated text. The improvement is especially visible in the minority class “yes” which indicates that fine-tuning helps the model better identify explicit symptom mentions that were often missed in the zero-shot setting. Despite the overall improvement, the results also suggest several possible directions for improving the fine-tuning setup. In this study, class imbalance was addressed using simple random oversampling, which effectively duplicates the limited number of “yes” and “no” examples. While this helps to balance class frequencies, it does not increase the diversity of training signals and may therefore limit the model’s ability to generalize to new expressions of symptoms. More advanced data augmentation strategies, such as synthetic generation of additional training examples, could potentially provide more diverse and informative training data and further improve performance. In addition, in this study, a single LoRA adapter was trained across all 53 QoL questions. While this approach is computationally efficient, it may limit the model’s ability to specialize for different types of questions, which vary in symptom type and linguistic formulation. Training separate adapters for different subsets of questions, or even per-question adapters, could potentially improve performance by allowing more targeted adaptation. However, such approaches would significantly increase computational cost and were beyond the scope of this study.

The analysis of textual evidence demonstrated that the tested model could successfully justify its predictions with supporting text. In nearly 90% of correctly classified yes and no cases, model-generated evidence overlapped with human-annotated spans, either exactly or partially. This finding is particularly relevant for clinical interpretability but also highlights the potential of LLMs to generate synthetic annotations that align with human annotations, which could reduce manual effort and increase the availability of labeled data for optimization methods that benefit from larger training samples.

Error analysis revealed systematic weaknesses related to mapping free-text symptoms description to discrete questionnaire items. Most misclassifications occurred when the model inferred an answer despite the absence of explicit information in the text, especially for a fallback question “Did you feel ill or unwell?” which exhibited a notably higher error rate (26%) compared to other questions. This is likely related to the semantic role of this label in the annotation scheme, as it functions as a broad catchall category for cases that do not match more specific symptom questions, as well as for general statements of wellness or illness [13]. As such, it often captures semantically underspecified or heterogeneous expressions, which may be inherently more difficult to map to a single EORTC QoL construct. Symptoms such as fever or low blood pressure did not fit into other predefined categories and were therefore annotated with this fallback label. However, for a model performing question-wise prediction, it is not clear whether a more specific matching category exists. Emotional and symptom-related questions with overlapping semantics (eg, pain vs discomfort, fatigue vs need for rest) were particularly prone to error. These findings highlight the challenge of defining clear decision rules for assigning free-text expressions to specific QoL questionnaire items. Even for human annotators, it may be difficult to determine whether indirect expressions (eg, *“*I constantly needed to rest”) should be interpreted as evidence for the question (“Were you tired?”) or whether only explicit mentions (eg, “I felt very tired”) should be considered correct. Overlapping questions, such as general worry and worry about health in the future, further complicate the task, as answering both questions positively may introduce redundancy, while restricting the answer to only one requires nuanced rules that are not defined in the questionnaire structure. These findings are consistent with Schmidt et al [13], who reported similar difficulties during the annotation process of the dataset used in this study.

### Comparison With Prior Work

A foundational work directly related to this research is that of Schmidt et al [13], which introduced a feasibility study for extracting QoL information from cancer forum posts from Inspire.com and comparing it to patient-reported outcomes on the EORTC QLQ-C30 and EORTC QLQ-BR23 questionnaires. This study demonstrated that QoL information is present in online forum posts and that manual coding can reliably link free-text content with structured questionnaire responses. However, it did not explore the potential of using AI to automate the process.

A systematic review by Lázaro et al [50] synthesized the scientific evidence on the application of NLP techniques as a QoL analysis tool in patients with chronic conditions, including cancer. However, this review covered studies published between 2011 and 2021 and therefore did not include recent advances in NLP which are part of this work. Individual studies mentioned in the review have addressed related but narrower tasks, such as detecting depressive symptoms in online forums (Karmen et al [51]), extracting patient-reported breast cancer symptoms from free-text electronic health record notes (Forsyth et al [21]), and detecting and quantifying pain in radiation oncology office notes of patients with cancer with bone metastases (Naseri et al [52]). While these studies demonstrate the feasibility of extracting QoL-related information from unstructured text, they focused on a specific subset of symptoms and did not attempt to map them to standardized QoL questionnaires.

More recently, researchers have begun to evaluate using LLMs for structured information extraction in clinical context, although with different task scopes and data sources than this study. For example, Garcia-Carmona et al [22] assessed the performance of 6 LLMs (both proprietary and open-source) in a zero-shot setting in extracting patient demographics, diagnostic details, and pharmacological data from unstructured medical reports. Lee et al [23] compared the performance of human reviewers and a locally deployed LLM for extracting key thyroid cancer histologic and staging information from surgical pathology reports. While these studies explore the potential of using LLMs for clinical NLP tasks, they focus on texts authored by medical professionals rather than patient-generated text and do not address QoL information.

A closely related study by Nair et al [24] evaluated multiple LLMs and prompting strategies for summarizing patient-generated content from web-based forums and health communities related to breast cancer. The authors compared zero-shot, few-shot, and CoT prompting results with manual reference summaries. They found that few-shot prompting outperformed other approaches, which aligns with observations from this study. However, summarization tasks differ fundamentally from extracting information to answer standardized questionnaire items.

In contrast to existing work, this study provides a systematic evaluation of open-source LLMs and optimization strategies for extracting questionnaire-aligned QoL information from patient-generated forum posts, using a breast cancer forum as an example. Unlike prior research focusing on different tasks, clinical text, or narrow symptom extraction, this study evaluates prediction accuracy for all symptoms included in EORTC QLQ-C30 and EORTC QLQ-BR23 questionnaires. Additionally, it investigates the potential of using LLMs for recreating human annotations by extracting text spans supporting the prediction. These contributions support the potential use of open-source LLMs in a real-world clinical setting for low-burden, interpretable QoL information extraction from patient-generated text.

### Limitations

This study has several limitations that readers should be aware of when interpreting the results. First, the dataset was derived from a single online breast cancer forum and included a limited number of patients and posts. Additionally, all participants were US residents, as Inspire.com members are primarily US-based. As a result, the findings may not be generalizable to other diseases or overall patient populations, where language use and symptom reporting may differ. Different patient communities may vary in how explicitly or implicitly symptoms are described and in how medical terminology is used, which may affect the mapping between forum posts and EORTC QoL measures. Future studies should assess whether similar performance patterns hold across other diseases, languages, and online platforms. This is especially important for non-English or culturally distinct forums, where symptom expression and linguistic framing may differ substantially and may not align with the assumptions embedded in EORTC-based labeling schemes.

Second, ground-truth labels were derived from manual annotations that required mapping patient-generated forum content to structured questionnaire items. As discussed earlier, some texts are overlapping or ambiguous, which poses difficulties even for human annotators. The mean Fleiss’ kappa for the data used in this study was 0.5, indicating moderate to high inter-annotator agreement. This remaining uncertainty may have affected the quality of the dataset and, consequently, both model performance estimates and error analyses.

Third, the dataset was restricted to posts where the author describes their own condition. Posts referring to other individuals (eg, relatives or friends) were excluded to maintain a clear definition of the target variable (QoL of the post author). While this improves label consistency, it artificially simplifies the task. In real-world applications, systems would need to first identify whether a post refers to the author or to another person before applying QoL classification.

Furthermore, this study focused exclusively on locally hosted, open-source LLMs. While this reflects realistic constraints in clinical settings due to privacy concerns, it limits direct comparison with proprietary models that may achieve different performance levels. In addition, only a subset of prompting and fine-tuning strategies was explored, and hyperparameters were not exhaustively tuned. All optimization experiments were conducted using a single model (GPT-OSS-20B) for controlled comparison, but the findings may not generalize to other families and sizes.

Finally, parameter-efficient fine-tuning was conducted using a single LoRA adapter shared across all 53 QoL questions. This design choice, made for computational efficiency, may limit the ability of the model to specialize across question types.

### Conclusions

In conclusion, this study demonstrates that extracting QoL information from patient-generated health care forum posts using open-source LLMs is a challenging task with limited and highly variable performance. Without task-specific training, baseline zero-shot models perform poorly. They consistently struggle to detect explicit symptom information (“yes” and “no” responses), which is the primary objective of this extraction task, and instead largely default to the majority “not in the text” class.

We found that prompt-based optimization strategies provide little to no benefit over zero-shot baselines. Parameter-efficient fine-tuning with LoRA proved significantly more effective, achieving the highest overall accuracy and better adapting to the patient-generated text. However, performance on the minority classes remains limited even after fine-tuning. Because missing an explicit symptom report in a real-world setting means failing to detect potential treatment side effects or emotional distress, automated QoL extraction with open-source LLMs is not feasible for clinical deployment. While fine-tuning offers a promising path forward, future work must focus on ensuring the reliable detection of minority-class signals.

## Abbreviations

bfloat16: brain floating point with 16 bits
CoT: Chain-of-thought
DSPy: Declarative Self-improving Python
EORCTC QLQ-BR23: 23-item European Organization for Research and Treatment of Cancer Quality of Life Questionnaire - Breast Cancer Module
EORTC: European Organization for Research and Treatment of Cancer
LLM: large language model
LoRA: low-rank adaptation
MIPROv2: Multiprompt Instruction Proposal Optimizer Version 2
NLP: natural language processing
QLQ-C30: Quality of Life Questionnaire-Core
QoL: quality-of-life
RQ: research question
